# Effect of Solution and Aging Temperatures on Microstructure and Mechanical Properties of 10Cr13Co13Mo5Ni3W1VE(S280) Steel

**DOI:** 10.3390/mi12050566

**Published:** 2021-05-17

**Authors:** Jinyan Zhong, Zun Chen, Shanglin Yang, Songmei Li, Jianhua Liu, Mei Yu

**Affiliations:** School of Materials Science and Engineering, Beihang University, Beijing 100191, China; chenzunzun@buaa.edu.cn (Z.C.); yslx001@buaa.edu.cn (S.Y.); songmei_li@buaa.edu.cn (S.L.); liujh@buaa.edu.cn (J.L.); yumei@buaa.edu.cn (M.Y.)

**Keywords:** 10Cr13Co13Mo5Ni3W1VE(S280), steel, mechanical properties, microstructure

## Abstract

The article investigated the effects of solution and ging temperatures on microstructure and mechanical properties of ultra-high strength stainless steel 10Cr13Co13Mo5Ni3W1VE(S280). Higher solution temperatures can improve impact toughness because of the quantity reduction of submicron-sized particles which act as microporous nucleation sites. S280 has the best mechanical properties at 1080 °C solution temperature. After quenching, the steel is completely martensite with almost no retained austenite. Aging at 560 °C results in peak strength due to the precipitation of fine carbides coherent zones. The loss of precipitates/matrix coherency and precipitates coarsening cause a decrease in strength at higher aging temperatures. Good strength and toughness obtained at 540 °C aging temperature are attributed to fine and dispersed strengthening phases such as Cr_2_C and Fe_2_Mo, and the recovery of austenite in high-density dislocation martensite matrix. The details of electron microscopy research, strengthening and toughening mechanisms are discussed.

## 1. Introduction

With the development of the aviation industry, the demand for high strength and toughness structural steels has promoted the progress of ultra-high strength stainless steel. The steels are designed for main load-bearing components of aircraft, such as landing gear and gas turbine engine shaft requiring high strength, high fracture toughness, and specific resistance to stress corrosion cracking and fatigue [1]. 

The high strength steels currently using for landing gear are traditional ultra-high strength steels, such as 4340, D6AC, 300M, GC-4, 35NCD16, 30CrMnSiNi_2_A [2,3,4,5,6,7,8] and AerMet100 [9]. However, they are restricted for use in marine atmospheres and corrosive environments due to poor corrosion resistance.

The common stainless steels such as 15-5PH and PH13-8Mo are limited in application because of low strength [10,11,12,13,14,15,16]. After years of research, components design, double vacuum melting, forging billet lumber and other key processes and technology of ultra-high strength stainless steel are developing [17,18]. 

A new ultra-high strength stainless steel 10Cr13Co13Mo5Ni3W1VE(S280) [19] has been developed successfully. Compared with AerMet100 and 300M, it has better corrosion resistance, higher strength (*σ*_b_ > 1900 MPa) and fracture toughness (*K*_IC_ > 90 MPa m^1/2^). The microstructure is supersaturated lath martensite after quenching and cold treatment. Aging at higher temperatures makes the strengthening phase precipitate on the lath martensite matrix. While having excellent corrosion resistance, S280 also has great strength and toughness. However, the microstructural features of S280 are still not understood. Therefore, this research aims to perform a detailed microstructural analysis of S280 to investigate the changes in mechanical properties during the aging process. Transmission electron microscopy (TEM) was used to characterize the strengthening carbides and austenite.

## 2. Materials and Methods

### 2.1. Material Details

The composition of S280 in this research is (mass fraction, %) Cr 13, Co 13, Mo 5, Ni 0.3 and W 0.1. The steel was forged into 150 mm rounds after melted by Vacuum Induction Melting/Vacuum Arc Remelting (VIM/VAR).

### 2.2. Heat Treatment Process

The billets were austenitized at 1080 °C for 1 h, then air-cooled and aged at 680 °C for 6 h. The samples for annealing treatment were heated to 900 °C, 1000 °C, 1040 °C, 1080 °C, 1120 °C and 1200 °C for 1 h, then oil quenched, refrigerated at −73 °C for 2 h and aged at 540 °C for 4 h. While the samples for aging treatment, the billets were austenitized at 1080 °C for 1 h, then oil quenched to room temperature, and immediately transferred to a cryogenic bath maintained at −73 °C. One was saved and others were severally aged at 100 °C, 200 °C, 300 °C, 400 °C, 450 °C, 500 °C, 520 °C, 530 °C, 535 °C, 540 °C, 545 °C, 550 °C, 555 °C, 560 °C, 600 °C, 650 °C and 700 °C for 4 h. 

### 2.3. Test Methods

The 10 mm diameter tensile samples, 10 × 10 × 55 mm impact toughness samples and 15 mm thick fracture toughness samples were machined from heat treatment billets in L-T orientation, then finishing. Tensile tests were measured at room temperature according to ASTM E8-91 at a 10^−4^/s strain rate by MST 810 Universal Testing Machine(Eden Prairie, MN, USA). The fracture toughness *K*_IC_ was measured by pre-cracked (a/w = 0.45–0.55) three-point samples according to ASTM E399-09.

Most of the samples were examined by optical microscopy (OM). For optical microscopy, the samples were etched in saturated CuCl_2_ solution(V_HNO3_:V_HCl_ = 3:1). The fracture cross section and composition of samples were observed by scanning electron microscope (SEM, FEI Nova Nano SEM 430) with energy dispersive spectroscopy (EDS). Selected samples were examined by transmission electron microscopy (TEM, HITACHI H-800 and JEOL 2010). For electron microscopy, the samples were manually grinded to a 0.05 mm thickness on emery paper, then electropolished in a chromium–acetic acid solution to make it thin. Subsequently, electropolished by a twin-jet machine in a mixture solution of CH_3_CH_2_OH and HClO_4_ (V_CH3CH2OH_:V_HClO4_ = 9:1) at −20 °C with applied potential of 40 V and current of 90–100 mA. For further analysis of the precipitated phase at the nanoscale, lattice image observation was carried out on a JEM-2010 high-resolution electron microscope. The accelerating voltage was 200 kV, the instrument constant was 15.6 MMA, and the point resolution of the electron microscope was 1.94 a. Images were filtered and regenerated by Fourier transform using digital micrograph software. Fourier transform was first performed on selected areas to obtain corresponding maps, which were calibrated with high resolution transmission electron microscopy (HRTEM) using matrix analysis to determine the structure, type and crystallographic orientation relationship of the precipitated phases. The phase composition of samples before and after aging was analyzed by XRD. The target material for XRD was copper, the scan range and rate for XRD measurement were 30–80°and 4°/min, respectively.

## 3. Results and Discussion

### 3.1. Effect of Solution Temperature on Mechanical Properties

**Figure 1:**
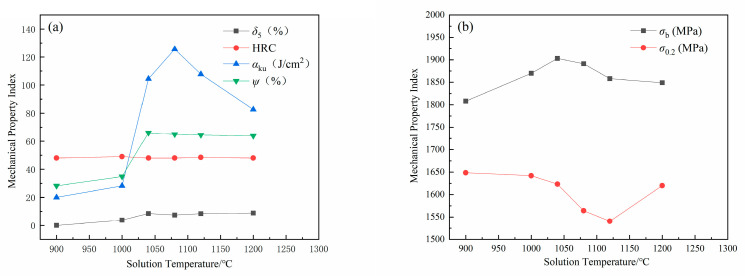
Effects of solution temperature on mechanical properties of S280 steel (**a**) elongation (*δ*_5_), reduction in area (*ψ*), impact toughness (*α*_ku_) and hardness (HRC) and (**b**) tensile strength (*σ*_b_), yield strength (*σ*_0.2_).

The tensile strength (*σ*_b_), yield strength (*σ*_0.2_), elongation (*δ*_5_), reduction in area (*ψ*), impact toughness (*α*_ku_) and hardness (HRC) at different solution temperatures are shown in Figure 1. Tensile strength increases from 1820 to 1920 MPa while the solution temperature raises from 900 °C to 1050 °C. Tensile strength decreases slowly when the solution temperature exceeds 1100 °C. Elongation and reduction change slightly when solution temperature increases from 900 °C to 1200 °C. Impact toughness increases while the solution temperature increases from 900 °C to 1080 °C, it reaches a maximum at 1080°C and then decreases. Above all, S280 has optimum mechanical properties at 1080°C solution temperature.

### 3.2. Effect of Solution Temperature on Austenite Grains

**Figure 2:**
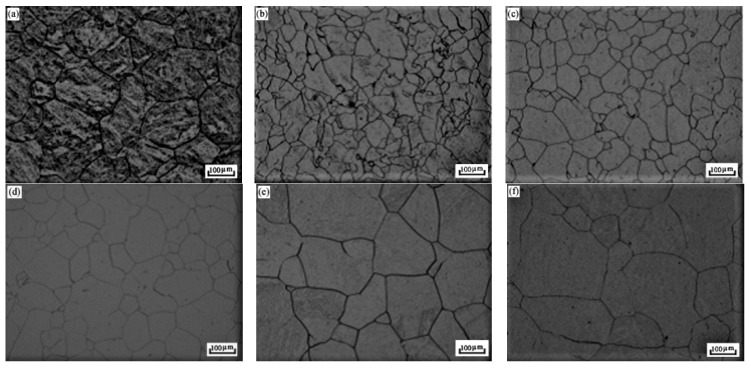
Optical photomicrographs show the effect of solution temperature on austenite grain. (**a**) 900 °C; (**b**) 1000 °C; (**c**) 1040 °C; (**d**) 1080 °C; (**e**) 1120 °C; (**f**) 1200 °C.

Figure 2 shows austenitic grains of S280 at different solution temperatures. The structure is further coarsened with the solution temperature increasing (Figure 2f). According to initial research and the dissolution of some alloy carbides, the optimum solution temperature is between 1000 °C and 1100 °C. The temperature chosen was 1080 °C because it is high enough to promote excess carbide solid solution and low enough to maintain a relatively fine grain structure. With the increase of solution temperature, the carbide particles at the grain boundary decreased gradually, while the austenite grain size increases due to the lack of pinning effect of carbide at the grain boundary. The large particles precipitated phase migration was blocked at the interface. With the decrease of grain boundary carbides, the grain boundary becomes flat. When the solution temperature is below 1080 °C, the carbides at the interface inhibit the high-temperature growth behavior of austenite grains.

### 3.3. Effect of Aging Temperature on Mechanical Properties

**Figure 3:**
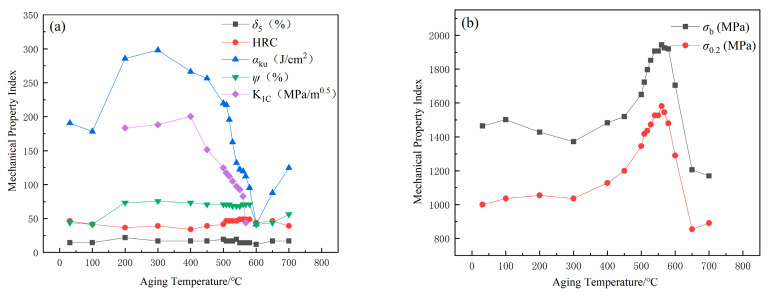
Effects of aging temperature on mechanical properties of S280 steel. (**a**) elongation (*δ*_5_), reduction in area (*ψ*), impact toughness (*α*_ku_), fracture toughness (*K*_IC_) and hardness (HRC) and (**b**) tensile strength (*σ*_b_), yield strength (*σ*_0.2_).

The effect of 4 h aging at 1080°C solution temperature of S280 is shown in Figure 3. Aging over a range of temperatures results in significant changes in mechanical properties which include tensile strength (*σ*_b_), yield strength (*σ*_0.2_), fracture toughness (*K*_IC_), elongation (*δ*_5_), area reduction (*ψ*), impact toughness (*α*_ku_) and hardness (HRC). From 100 °C to 300 °C, tensile strength increases first, then slowly decreases, and rapidly increases above 300 °C. Tensile strength and hardness peak at 560 °C. Tensile strength decreases rapidly above 560 °C and its trend is roughly the same with yield. Impact toughness shows a minimum at 100 °C and a substantial increase occurs at 300 °C. The aging above 550 °C causes impact toughness to drop rapidly. Fracture toughness increases first but then decreases slowly from 400 °C to 600 °C. S280 has high toughness but relatively low strength from 200 °C to 300 °C. The optimum combination of tensile strength and toughness is obtained between 540 °C and 550 °C.

### 3.4. Effect of Aging Temperature on Optical Microstructure

**Figure 4:**
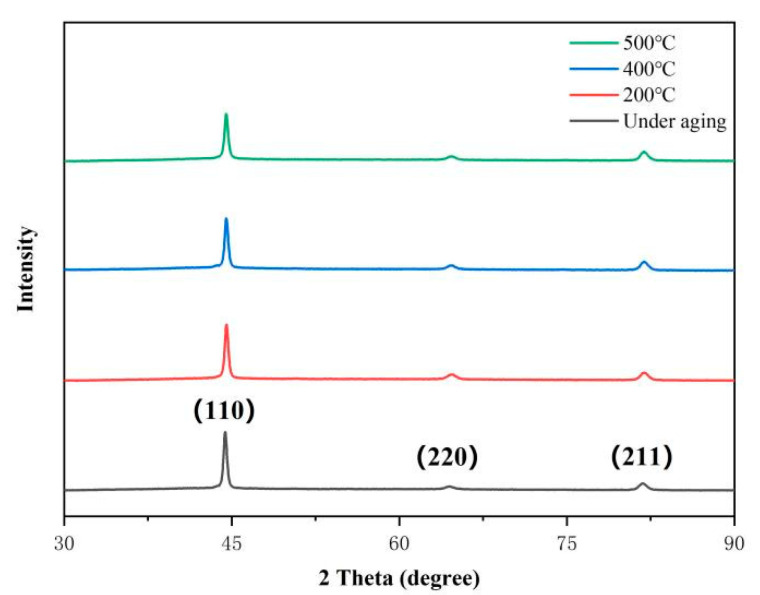
XRD image of the samples aged at different temperatures.

The XRD image of S280 at different aging temperatures is shown in Figure 4. The XRD result shows that there is no obvious phase change before and after aging. There is no obvious peak of austenite in this figure. This shows that the microstructure of the sample is martensite when the solution temperature is 1080 °C, and the aging process is the process of refining martensite lath.

**Figure 5:**
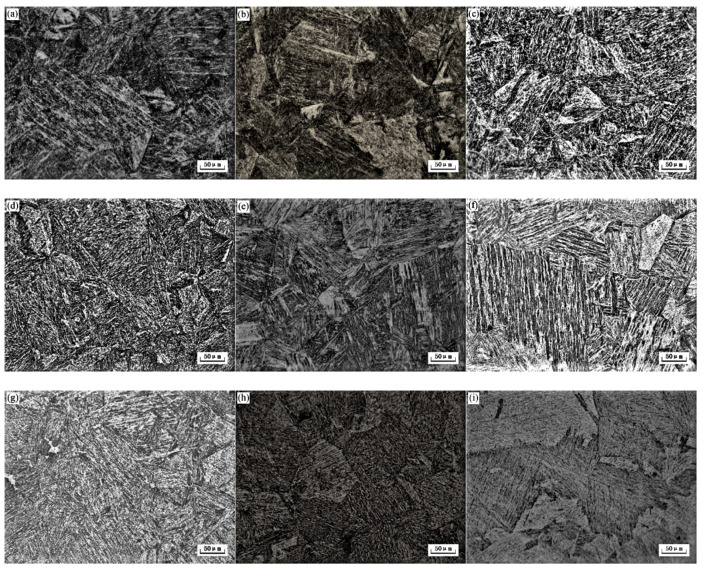
Optical photomicrographs show the effect of aging temperature on optical microstructure. (**a**) Under aging; (**b**) 200 °C; (**c**) 500 °C; (**d**) 535 °C; (**e**) 540 °C; (**f**) 560 °C; (**g**) 600 °C; (**h**) 650 °C; (**i**) 700 °C.

The microstructures of S280 at different aging temperatures are shown in Figure 5. The matrix structure is lath martensite whose size changes significantly under different aging conditions. As aging temperature increases, the size of lath martensite does not increase or decrease monotonically but has a complicated trend. S280 obtained a relatively small and ideal lath martensite at 540°C (Figure 5e), the width is about 5 to 10 μm and the length is about 50 to 100 μm.

### 3.5. Effect of Aging Temperature on TEM Microstructure

**Figure 6:**
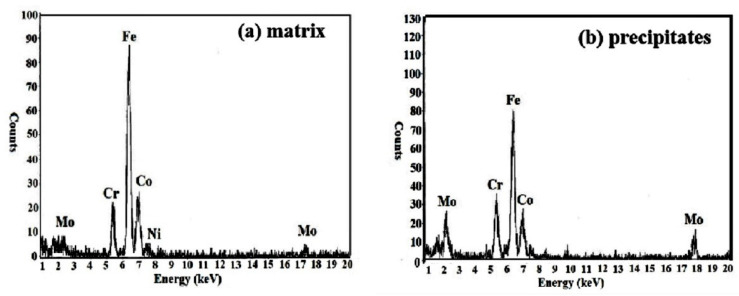
EDS Spectra of the sample aged at 540°C. (**a**) matrix; (**b**) precipitates.

The EDS results of the sample aged at 540 °C are shown in Figure 6. It can be seen that the contents of element Cr and Mo in precipitates are significantly higher than that in matrix, indicating that the precipitates may be the compounds of Fe and Cr or Fe and Mo. 

**Figure 7:**
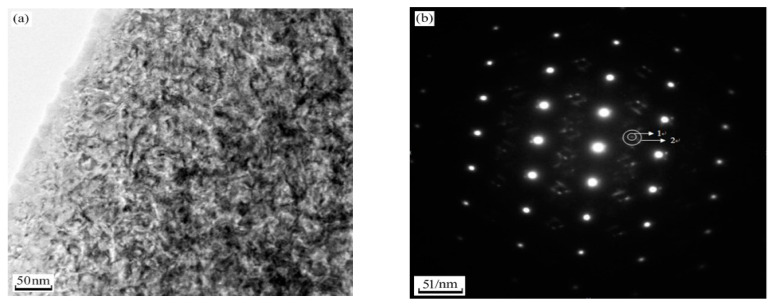

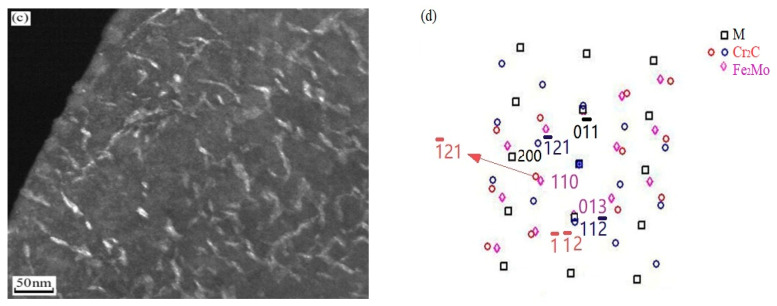
Sample aged at 540 °C with an orientation [011] matrix. (**a**) Bright-field image shows the precipitates; (**b**) electron diffraction pattern from the precipitates shown in (**a**); (**c**) corresponding dark-field image; (**d**) schematic indexing of the pattern from (**b**).

The microstructure of 540 °C aged sample was observed by high-resolution electron microscopy in the direction of incident electron beam along the martensite [011]. In martensitic matrix, two kinds of extremely fine, needle-shaped precipitates are observed and the crystal structure of them could be identified by electron diffraction analysis (Figure 7). Figure 7a is a typical bright-field image obtained close to a [011] _M_ orientation. Most precipitates exhibit strain contrast, this indicates that they still maintain coherency with the matrix. The selected area diffraction pattern is shown in Figure 7b. Only some of the precipitates show patterns that could be indexed as the Cr_2_C carbides with orthorhombic structure, the crystal lattice constant is a = 0.4884 nm, b = 0.5599 nm, c = 0.4438 nm. Another precipitate is Fe_2_Mo with the hexagonal structure, the crystal lattice constant is a = b = 0.473 nm, c = 0.772 nm. These are relatively common in ultra-high strength steel and the results of TEM are consistent with EDS. The universal relationship of Cr_2_C and Fe_2_Mo can be expressed by [20]:

$${\left(1\bar{1}0\right)}_{M}//{\left(\bar{1}2\bar{1}\right)}_{Cr2C},\;{\left[111\right]}_{M}//\;{\left[111\right]}_{Cr2C}$$

$${\left(110\right)}_{M}//{\left(\bar{1}2\bar{1}\right)}_{Cr2C},\;{\left[1\bar{1}\bar{1}\right]}_{M}//{\left[111\right]}_{Cr2C}$$

$${\left(\bar{1}2\bar{1}\right)}_{M}//{\left(001\right)}_{Fe2Mo},\;\;{\left[111\right]}_{M}//\;{\left[100\right]}_{Fe2Mo}$$

**Figure 8:**
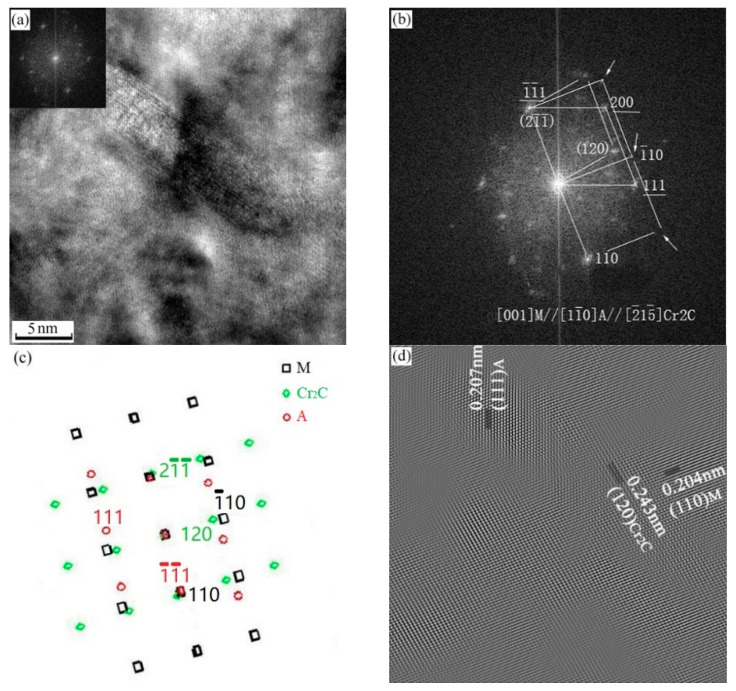
Sample aged at 540 °C with an orientation [001] matrix. (**a**) high resolution electron image; (**b**) electron diffraction pattern from the precipitates shown in (**a**); (**c**) schematic indexing of the pattern from (**b**); (**d**) filter image.

The aging precipitates are relatively small and the appearance is spherical or ellipsoidal. The size is about several tens of nanometers (Figure 8c,d).

The bright-field images show the formation of austenite at plate boundaries in film morphology (Figure 8a). The thickness of austenitic film is small, about 3 nm, and it cannot be determined whether it is formed by nucleation and growth or by reverse shear. The universal expression through calibration is given by:

$${\left(011\right)}_{M}//\;{\left(\bar{1}2\bar{1}\right)}_{Cr2C},\;{\left[\bar{1}1\bar{1}\right]}_{M}//{\left[111\right]}_{Cr2C}$$

$${\left(110\right)}_{M}//\;{\left(\bar{1}\bar{1}1\right)}_{A},\;{\left[1\bar{1}\bar{1}\right]}_{M}//{\left[011\right]}_{A}$$

**Figure 9:**
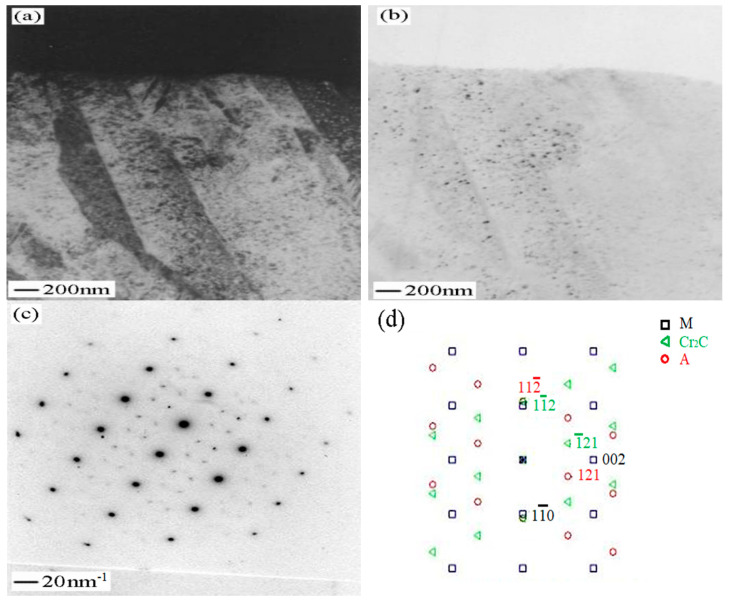
Sample aged at 560 °C. (**a**) Bright−field image showing Cr_2_C precipitates; (**b**) corresponding dark-field image; (**c**) electron diffraction pattern from Cr_2_C precipitates shown in (**a**); (**d**) schematic indexing of the pattern from (**c**).

The bright-field image and corresponding dark-field image showing Cr_2_C precipitates; (b) appear in the diffraction pattern after aging at 560 ℃ (Figure 9). Schematic indexing of the pattern shows $\left(1\bar{1}0_{M}\right)$//${\left(11\bar{2}\right)}_{Cr2C}$, $\left[110_{M}\right]$//${\left[513\right]}_{Cr2C}$ (Figure 9d). The transformation matrices A and B of surface and direction index between martensite and Cr_2_C can be calculated. The surface index and the direction index of the matrix and the precipitated phase are known. Using the matrix calculator, an invertible matrix can be calculated so that the vector obtained by multiplying the matrix with the exponential vector of the precipitated phase is equivalent to the exponential vector of the matrix.

$$\left[\begin{array}{c} h\\ k\\ l \end{array}\right]=A^{-1}\left[\begin{array}{c} h\prime \\ k\prime \\ l\prime \end{array}\right]=\left[\begin{array}{c} 1.494\;\;\;\;\;\;\;\;\;0.564\;\;\;\;\;\;\;\;0.555\\ 0.735\;\;\;\;-0.195-1.782\\ -0.420\;\;\;\;\;\;1.439\;\;\;-0.331 \end{array}\right]\left[\begin{array}{c} 1\\ \bar{1}\\ 0 \end{array}\right]\sim \left[\begin{array}{c} 1\\ 1\\ \bar{2} \end{array}\right]$$

$$\left[\begin{array}{c} u\\ v\\ w \end{array}\right]=B^{-1}\left[\begin{array}{c} u\prime \\ v\prime \\ w\prime \end{array}\right]=\left[\begin{array}{c} 0.523\;\;\;\;\;\;\;\;0.197\;\;\;\;\;\;\;\;\;\;\;\;0.194\\ 0.196\;\;-0.052\;\;\;\;\;-0.475\\ -0.178\;\;\;\;\;0.610\;\;\;\;\;-0.140 \end{array}\right]\left[\begin{array}{c} 1\\ 1\\ \bar{1} \end{array}\right]\sim \left[\begin{array}{c} 1\\ 1\\ 1 \end{array}\right]$$

where <*hkl*> is the surface index of the matrix, <*h′k′l′*> is the surface index of precipitated phase, <*uvw*> is the direction index of the matrix, and <*u′v′w′*> is the direction index of precipitated phase.

It is inferred that one of the 12 equivalent variants in general expression of M-Cr_2_C orientation relation consistent with Figure 6 is: ${\left(1\bar{1}0\right)}_{M}$//${\left(11\bar{2}\right)}_{Cr2C}$, ${\left[11\bar{1}\right]}_{M}$//${\left[111\right]}_{Cr2C}$. Cr_2_C is needle-shaped and uniformly distributed on dislocations, of which length or diameter range is from a few to a dozen nanometers.

**Figure 10:**
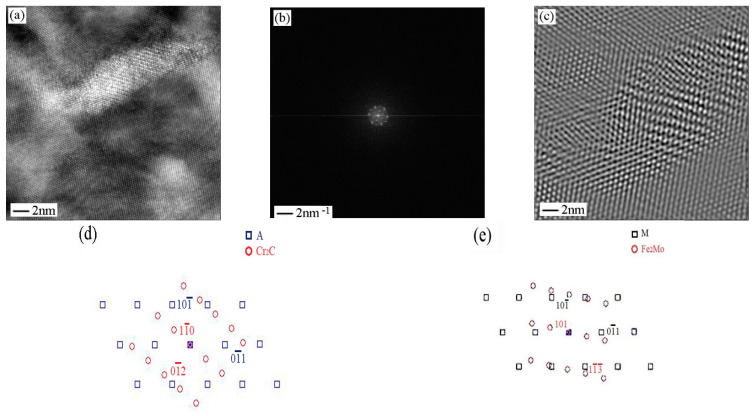
Sample aged at 560 °C. (**a**) High resolution electron image shows Cr_2_C and Fe_2_Mo precipitates; (b) electron diffraction pattern from the Cr_2_C and Fe_2_Mo precipitates shown in (**a**); (**c**) filter image; (**d**) Cr_2_C phase schematic indexing of the pattern from (**b**); (**e**) Fe_2_Mo phase schematic indexing of the pattern from (**b**).

Figure 10 is a high-resolution electron image of strengthening phase Cr_2_C and Fe_2_Mo. The diffraction spots of Figure 10b are obtained by FFT transformation from Figure 10a. After the calibration, directions of matrix, Cr_2_C and Fe_2_Mo are [111], $\left[\bar{221}\right]$ and $14\bar{1}$ respectively. The lattice image in Figure 10c is obtained by FFT transformation of diffraction spots. It can be seen that Cr_2_C (length is 12 nm, width is 4 nm) and Fe_2_Mo (length is 20 to 30 nm, width is 5 nm) retain a coherent lattice relationship with matrix and there is a certain degree of mismatch at the interface of coherent lattice.

**Figure 11:**
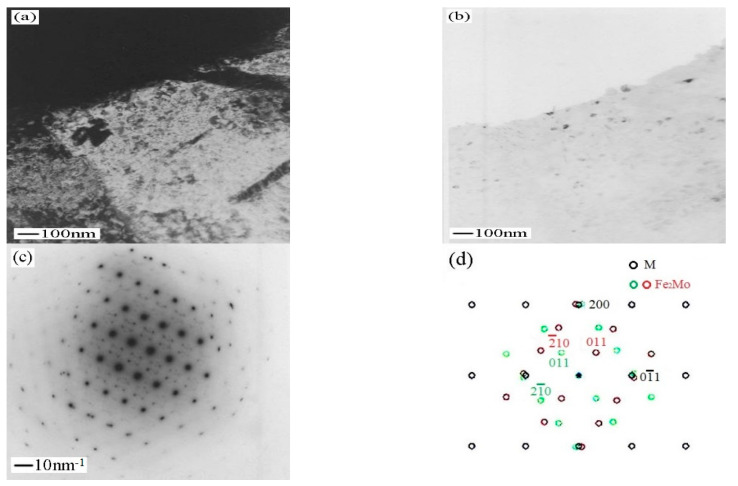
Sample aged at 560 °C. (**a**) bright-field image shows Fe_2_Mo precipitates; (**b**) corresponding dark−field image; (**c**) electron diffraction pattern from Fe_2_Mo shown in (**a**); (**d**) schematic indexing of the pattern from (**c**).

The precipitated phase Fe_2_Mo is ellipsoid dispersively distributed in matrix (Figure 11). Compared with Cr_2_C, Fe_2_Mo has a slightly larger diameter and a smaller volume fraction. The schematic index for this pattern shows ${\left(0\bar{1}1\right)}_{M}$//${\left(\bar{2}01\right)}_{Fe2Mo}$, ${\left[011\right]}_{M}$//${\left[\bar{12}2\right]}_{Fe2Mo}$ (Figure 11d). The transformation matrices A and B of surface and direction index of martensite and Fe_2_Mo are obtained

$$\left[\begin{array}{c} h\prime \\ k\prime \\ l\prime \end{array}\right]=A\left[\begin{array}{c} h\\ k\\ l \end{array}\right]=\left[\begin{array}{c} -0.512\;\;\;\;\;\;\;\;\;1.099\;\;\;\;\;\;\;\;1.099\\ 0.601\;\;\;\;\;\;\;\;-1.438\;\;\;\;\;\;0.499\\ 2.452\;\;\;\;\;\;\;\;\;\;\;\;\;1.055\;\;\;\;\;\;\;\;0.087 \end{array}\right]\;\left[\begin{array}{c} 0\\ \begin{array}{c} \bar{1}\\ 1 \end{array} \end{array}\right]\sim \left[\begin{array}{c} 0\\ 2\\ 1 \end{array}\right]$$

$$\left[\begin{array}{c} u\prime \\ v\prime \\ w\prime \end{array}\right]=B\left[\begin{array}{c} u\\ v\\ w \end{array}\right]=\left[\begin{array}{c} -0.105\;\;\;\;\;0.189\;\;\;\;0.671\\ -0.172-0.442\;\;\;\;0.522\\ 0.344\;\;\;\;\;\;\;\;0.148\;\;\;\;\;0.012 \end{array}\right]\left[\begin{array}{c} \bar{1}\\ \begin{array}{c} \bar{1}\\ \bar{1} \end{array} \end{array}\right]\sim \left[\begin{array}{c} \bar{3}\\ \begin{array}{c} \bar{1}\\ \bar{2} \end{array} \end{array}\right]$$

The general expression of M-Fe_2_Mo orientation relation consistent with Figure 11 is derived. One of the 12 equivalent variables is: (011)//$\left(02\bar{1}\right)$ and [111]//[312], which can be simplified to: ${\left(112\right)}_{M}$//${\left(001\right)}_{Fe2Mo}$ and ${\left[\bar{11}1\right]}_{M}$//${\left[100\right]}_{Fe2Mo}$.

**Figure 12:**
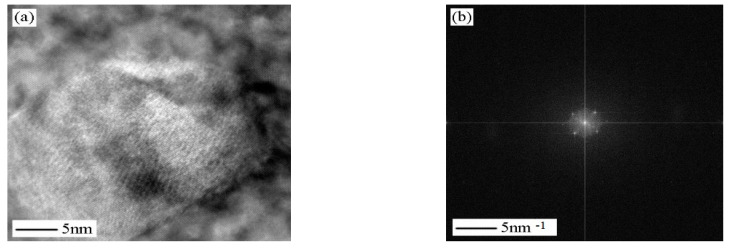

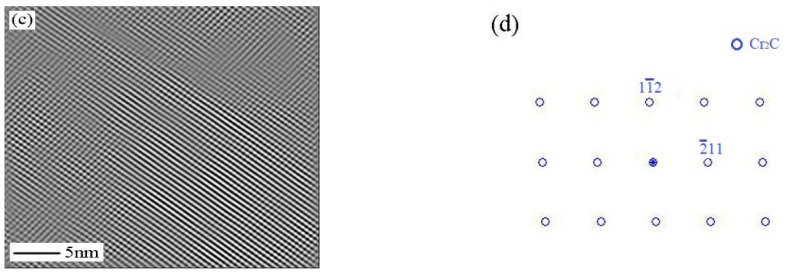
Sample aged at 600 °C. (**a**) High resolution electron image shows Cr_2_C precipitates in total loss coherency with matrix; (**b**) electron diffraction pattern from Cr_2_C shown in (**a**); (**c**) IFFT image; (**d**) schematic indexing of the pattern from (**b**).

Cr_2_C and Fe_2_Mo strengthening phases are observed at 600 °C aging temperature. Compared with aging at 560 °C, precipitated phase grows larger and becomes thicker, and the relationship between precipitated phase and matrix is non-coherent. Figure 12 shows a high-resolution electron image of non-coherent phase Cr_2_C. The diffraction spots of Figure 12b are obtained by FFT transformation from Figure 12a. After the calibration, the direction of precipitated phase is [351]. The lattice image in Figure 12c is obtained by IFFT transformation of diffraction spots. The length and width of Cr_2_C are about 27 nm and 20 nm.

**Figure 13:**
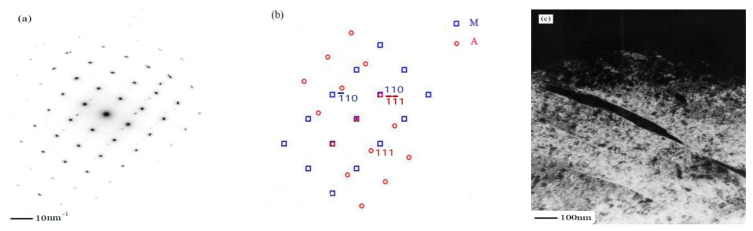
Sample aged at 600 °C with an orientation [001] matrix. (**a**) Electron diffraction pattern from the reverted austenite shown in (**c**); (**b**) schematic indexing of the pattern from (**a**); (**c**) dark−field image shows reverted austenite.

As well as precipitates coarsening, aging at 600 °C results in precipitate/matrix coherency losing and reverted austenite volume fraction coarsening. Figure 13a shows electron diffraction pattern from the reverted austenite close to a [001]_M_ orientation. An appreciable reversion of austenite is observed along plate boundaries; the needles are clearly seen (Figure 13c). The reverted austenite forms primarily at plate boundaries with a Kurdjmov–Sachs relationship. It is indicated that austenite is formed by nucleation and growth because it has no dislocation.

### 3.6. Influence of Microstructure on Strength and Toughness

Strength peak at 560 °C is a result of microstructure: tiny and irregularly shaped coherent zones or carbides have a uniform dispersion. The current research also indicates that peak intensity is achieved when precipitates are fully coherent with matrix, consistent with Ayer [9] and Olson [21]. The stress could concentrate around carbides and results in initiate cracks by decohesion of carbide/matrix interface, an appropriate distribution, shape and size of precipitated phase could provide high mechanical properties [22]. The aging above 560 °C makes the yield and tensile strength decrease. The results also show that quenched dislocation density retains peaking at 560 °C. This observation is consistent with the proposal of Speich [23]. Therefore, the strength of S280 is derived from high dislocation density and fine coherent precipitates dispersion.

**Figure 14:**
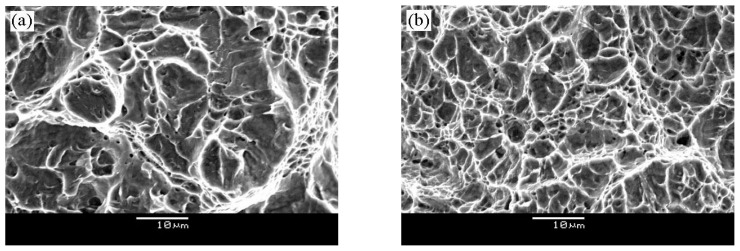
SEM micrographs of fracture toughness samples aged at (**a**) 450 °C and (**b**) 540 °C.

The research of toughness variation during aging indicates several important trends. The toughness of quenched steel is high because of low strength, absence of coarse inclusions and residual austenite at the plate boundary. Thomas first demonstrated the beneficial effect of retained austenite on toughness [24]. Aging at 540 °C, strength and toughness are well combined because of fine Fe_3_C, the formation of thin-film reverted austenite and the uniform precipitation of Cr_2_C. Its toughness is comparable to quenched and cryogenically treated steel, but the strength is much higher, so it has a higher strength/toughness ratio. The reverted austenite has a thin-film morphology which is similar to the retained austenite observed in quenched steels [24]. Coarsening of austenite at lath boundaries allows for some degree of lath boundary migration (i.e., recovery), however, a high-density of precipitates pin the lath boundaries during heat treatment [25]. It is now accepted that toughness can be improved only when austenite is present in film morphology and external stress or strain is applied [21]. The fracture morphology aged at 450 °C and 540 °C is nearly 100% microvoid coalescence which is the main fracture mechanism (Figure 14).

However, aging at 600 °C makes strength and toughness decrease because of precipitates coarsening (Figure 11). The austenite forming at this temperature is too coarse so that precipitates cannot pin lath boundaries [24]. The carbon content of austenite is expected to decrease when aged from 600 °C. Therefore, reverted austenite at higher aging temperatures will transform to martensite upon straining. Since the carbon content of this martensite is higher than average, the transformation during straining will impair toughness (Figure 3).

## 4. Conclusion

The relationship between microstructure and mechanical properties of S280 ultra-high strength stainless steel was obtained through experiments. In order to comprehensively consider the small size, light weight, and high strength of the part material, the study shows that:

1. The maximum strength in S280 was achieved when it aged at 560 °C for 4 h. This is attributed to the delayed dislocation recovery of the martensitic matrix and the formation of needle-shaped, coherent precipitates. A decrease in yield and tensile strength upon overaging results from precipitates/matrix coherency loss.
2. When aging at 540 °C, Cr_2_C, Fe_2_Mo and other fine and dispersed strengthening phases are uniformly precipitated and reverted austenite are distributed in a high-density dislocation martensitic matrix. These ensure good strength and toughness.
3. A higher solution temperature improves impact toughness because the number of sub-micron sized microvoid-nucleation particles reduces. A solution temperature of 1080 °C will provide the optimum mechanical properties for S280.

We will further functionalize its structure to evaluate its corrosion resistance, hydrogen embrittlement and stress corrosion sensitivity to ensure the long life and high reliability of aircraft parts.

## Data Availability

Not applicable.
